# An Efficient Agrobacterium-Mediated Transient Transformation System Using In Vitro Embryo-Derived Seedlings for Gene Function Elucidation in *Paeonia ostii*

**DOI:** 10.3390/plants14162498

**Published:** 2025-08-12

**Authors:** Yuhui Zhai, Xinrong Xie, Liping Zhang, Xuefei Wang, Zixuan Zhang, Lixin Niu, Yanlong Zhang

**Affiliations:** College of Landscape Architecture and Arts, Northwest A&F University, Yangling 712100, China; zhaiyuhui@nwafu.edu.cn (Y.Z.); xiexinrong@nwafu.edu.cn (X.X.); zlp0501@nwafu.edu.cn (L.Z.); wxuefei@nwafu.edu.cn (X.W.); 2024057025@nwafu.edu.cn (Z.Z.)

**Keywords:** *Paeonia ostii*, in vitro embryo-derived seedlings, Agrobacterium, transient transformation system

## Abstract

*Paeonia ostii* is an economically significant species serving as an ornamental, medicinal herb, and woody oilseed crop. Gene function elucidation and molecular breeding are hindered by the lack of efficient, stable transformation methods due to tissue culture challenges. To enable year-round functional studies without material constraints, we established a novel transient transformation system mediated by Agrobacterium using in vitro embryo-derived seedlings (TTAES) in *P. ostii*. By optimizing embryo germination media, we achieved consistent seedling production. Orthogonal experiments with a GUS reporter identified optimal conditions: OD_600_ = 1.0, 200 μM of acetosyringone, six negative-pressure treatments, and 2 h infection. Under this optimized system, maximum transformation efficiency was achieved at 35 days after germination. With this system, we demonstrated its application in investigating transcription factor-mediated regulation of target gene promoters using GUS as a reporter gene. To achieve non-destructive identification of transiently transformed plants, we employed GFP as a reporter gene. Using transient expression of VIGS (knockdown) and 35S constructs (overexpression), we characterized gene functions, thereby confirming the system’s effectiveness for functional analysis. This system facilitates the acquisition of plant experimental materials and significantly improves research efficiency for year-round gene function elucidation in *P. ostii*.

## 1. Introduction

Tree peony, a crop of significant economic importance that has been cultivated in China for over 2000 years [1], is highly prized for both its ornamental value and medicinal properties [2,3]. The recent advancements witnessed in the cut flower markets and seed oil production of peonies have further fueled growth within this sector [4,5]. The fleshy root bark of *Paeonia ostii* is a key source of Moutan Cortex in herbal medicine [6]. Meanwhile, *P. ostii* is recognized as a promising woody oilseed crop due to its high seed oil content and rich unsaturated fatty acid profile [7,8]. Following the completion of the chromosome-level genome assembly of *P. ostii* [9], research on the molecular mechanisms underlying genetic improvement has significantly expanded [10,11,12]. However, peony tissue culture remains plagued by persistent challenges, including severe explant browning, vitrification, contamination, difficulties in differentiation, limited regeneration efficiency, and suboptimal rooting [13,14]. Consequently, the lack of stable genetic transformation systems has made transient transformation crucial for elucidating gene functions and promoting molecular breeding in peony [15,16].

Transient transformation is a rapid and convenient approach to gene function analysis. Currently, the three predominant methodologies for transient genetic transformation in plants are gene gun, protoplast transformation, and Agrobacterium-mediated infiltration [17,18]. Among these techniques, Agrobacterium-mediated infiltration has been proven particularly efficacious due to its simplicity, speed, and efficiency in facilitating transient, high-level expression of target genes, thus making it the favored method for plant gene characterization [19]. This technique involves the introduction of suspension-cultured Agrobacterium cells into intact plant organs. Notably, the transformation efficiency of this technique is influenced by several parameters, including bacterial concentration (OD_600_), infiltration duration, and acetosyringone (AS) concentration [20]. Agrobacterium-mediated transient overexpression is widely used for gene function verification in peony [21]. Additionally, tobacco rattle virus (TRV)-mediated virus-induced gene silencing (VIGS) represents a commonly employed method for gene silencing analysis [10]. Agrobacterium-mediated delivery facilitates the introduction of TRV vectors into diverse plant tissues (e.g., buds, leaves, roots, and seeds) to achieve transient gene silencing [22,23,24,25]. However, a critical limitation is that fresh tissue materials required for these experiments are often seasonally restricted, such as being limited to specific growth stages. Consequently, elucidating gene function in peony takes significantly longer compared with model plants exhibiting year-round growth.

Besides their large quantity, *P. ostii* seeds are easy to preserve after harvest and show high germination rates [26,27]. However, under natural conditions, germination and rooting require approximately three months to complete, and the quality of open-field planted seedlings is unstable [28]. Embryo culture technology has been demonstrated to facilitate accelerated seedling development from *P. ostii* embryos, thereby reducing the breeding cycle and enhancing germination rates [29]. Nevertheless, this technique continues to encounter difficulties in terms of achieving optimal rooting efficiency. Given the short experimental duration of the transient transformation system, it remains to be determined whether transient transformation assays can be exclusively performed using in vitro embryo-derived seedlings without prior rooting. Recently, an Agrobacterium-mediated transient genetic transformation system has been established in vitro embryo-derived seedlings of *P. lactiflora* [30]. Nonetheless, the system investigated the transient transformation of *P. lactiflora* seedlings cultivated in vitro, neglecting to examine the growth conditions of in vitro embryo-derived seedlings. In addition, the transient transformation system developed for *P. lactiflora* relies solely on GUS labeling, which inflicts irreversible damage on plant materials following detection, thereby limiting its utility for in vivo studies [31]. The development of an efficient genetic transformation system for *P. ostii* (tree peony) presents distinct and significant challenges compared to *P. lactiflora* (herbaceous peony), primarily stemming from its woody perennial nature. Woody plant tissues, including seeds and embryos from *P. ostii*, are often more difficult to surface-sterilize effectively due to crevices and protective structures, leading to higher contamination risks in tissue culture compared to *P. lactiflora* [32,33]. Like many woody plants, *P. ostii* produces abundant secondary metabolites, such as paeonol, which exhibits antibacterial activity against diverse bacteria [34]. This increases the complexity of the Agrobacterium-mediated transient transformation system, a complexity that is generally less pronounced in herbaceous plants [35].

The present application of Agrobacterium-mediated transient genetic transformation in peonies is predominantly focused on gene transient overexpression and silencing. There has been little investigation into subcellular protein localization, protein–protein interactions, or transcription factor-target promoter relationships. It is imperative to acknowledge that, as a woody crop, *P. ostii* is distinct from the herbaceous crop *P. lactiflora*, and thus exhibits unique optimal germination and transient transformation requirements.

In this study, we established a novel transient transformation system mediated by Agrobacterium using in vitro embryo-derived seedlings in *P. ostii*. This system was designated as the transient transformation system mediated by Agrobacterium using in vitro embryo-derived seedlings (TTAES). By systematically screening embryo germination media, we achieved a consistent annual supply of experimental materials, overcoming seasonal limitations. Orthogonal experiments were conducted using the GUS gene as a reporter, with the objective of optimizing key parameters. The following factors were identified as the key elements for optimizing the Agrobacterium infection conditions: the bacterial concentration (OD_600_), AS concentration, negative pressure treatment frequency, and infection duration. The infection efficiency was then assessed at distinct developmental stages of axenic embryogenic seedlings under optimized conditions. Finally, the system was validated during the verification of the transcription factor-activated target gene promoter, and its applications in gene silencing and overexpression were discussed with GFP as a marker.

## 2. Materials and Methods

### 2.1. Plant Materials and Growth Conditions

**Figure 1:**
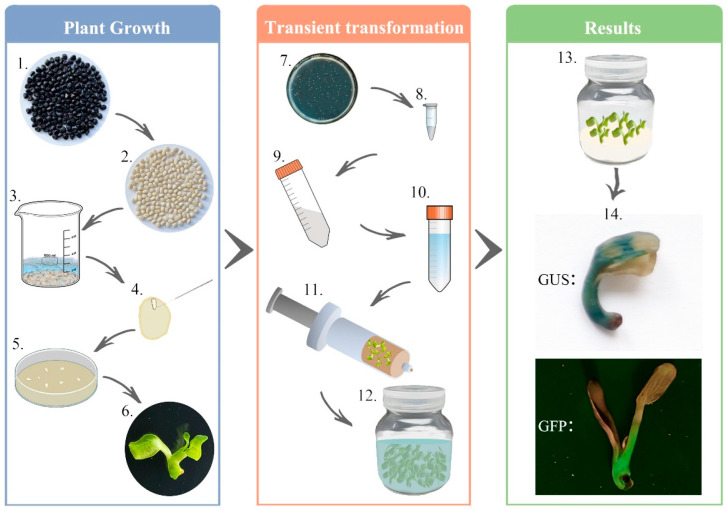
**Overview of TTAES protocol in *P. ostii*.** The protocol is divided into three sections: Plant Growth, Transient Transformation, and Results. Plant growth: (1) select large, plump seeds, then water selection and GA_3_ treatment; (2) remove the seed coat; (3) sterilize the seeds in a laminar flow cabinet; (4) isolate the seed embryos using a dissection needle; (5) inoculate the seed embryos onto embryo germination medium; (6) culture in a tissue culture chamber. Transient transformation: (7) transform the target gene vector into Agrobacterium GV3101; (8) inoculate a single colony into 200 µL of YEP liquid medium; (9) inoculate the bacterial culture into fresh YEP medium at a 1:100 dilution for a second round of cultivation; (10) collect the bacterial cells and resuspend them; (11) subject the embryogenic seedlings to vacuum infiltration using a syringe; (12) co-cultivate the vacuum-infiltrated seedlings with the resuspended bacterial solution at 28 °C, 100 rpm, in the dark. Results: (13) after air-drying the seedlings on sterile filter paper, inoculate them onto co-culture medium; (14) perform GUS staining or GFP fluorescence observation according to the tag carried by the vector.

*P. ostii* were cultivated in the Peony Resource Nursery of Northwest A&F University. Uniform-genotype seeds were produced by controlled cross-pollination between selected mother and father plants. Mature seeds were collected 120 days after artificial pollination and air-dried for use as experimental materials. A refined protocol for *P. ostii* seed embryo germination was applied as follows [14,30,36,37]: Large and plump seeds were selected and soaked in water for 48 h. Subsequently, seeds that sank to the container bottom were treated with a 1 g/L GA_3_ solution for 24 h. After removing the seed coat, seeds were rinsed repeatedly under tap water for 1 h, followed by half an hour in detergent water. Thereafter, they were rinsed again under tap water for 15 min. Seeds were then transferred to the benchtop, where they were disinfected with 75% ethanol for 30 s, washed twice with sterile water, treated with 2% sodium hypochlorite for 6 min, washed twice with sterile water, and subjected to a second 2% sodium hypochlorite treatment for 6 min, followed by six rinses with sterile water. Finally, seed embryos were excised using a scalpel and dissection needle and inoculated onto embryo germination media (Figure 1).

The embryo germination media used included Murashige and Skoog (MS) medium and Woody Plant Medium (WPM) [17,30]. WPM is characterized by lower overall nutrient levels—specifically reduced nitrogen and potassium—that may better suit woody plants’ growth requirements, and by relatively higher calcium content that may enhance cell wall stability and stress tolerance [38].MS medium: MS supplemented with 30 g/L sucrose, 0.5 mg/L 6-BA, and 1.0 mg/L GA_3_ (pH 5.6); WPM: WPM supplemented with 30 g/L sucrose, 0.5 mg/L 6-BA, and 1.0 mg/L GA_3_ (pH 5.6). Inoculated embryos were then cultured in an artificial growth chamber under a 16 h light (2000 Lx)/8 h dark photoperiod at 24 ± 2 °C. Embryo germination rates in both media were recorded every five days starting from 5 days after inoculation. The germination rate was calculated as (number of germinated seedlings/number of inoculated embryos) × 100%, with data collected at five-day intervals. Each medium was inoculated with 30 embryos, with three biological replicates per medium.

### 2.2. RNA Extraction and Gene Expression Profiling

In this experiment, total RNA extraction was conducted in accordance with the instructions provided in RNAprep Pure Plant Kit (TIANGEN, Beijing, China). Using the PrimScript^TM^RT reagent Kit with gDNA Eraser (Takara, Kusatsu, Shiga, Japan), synthesize first-strand cDNA. 18S-26S ITS RNA was used as the internal standard to assess mRNA abundances following the 2^−ΔΔCt^ method. RT-qPCR was performed using SYBR^®^ Premix Ex TaqTM II (Takara, Japan) on a StepOne Real Time PCR system. Primers used for vector construction and RT-qPCR are listed in Table 1.

### 2.3. Construction of Recombinant Plasmids

The cDNA from *P. ostii* seeds was used as the template, and primers 35S:PoABI5-F/R, PoABI5-pTRV2-F/R, and PoABI5-GFP-F/R were used for PCR amplification. The full-length CDS of PoABI5 was cloned into the pCAMBIA1300, pTRV2, and pCAMBIA2300-eGFP vectors using one-step cloning. Notably, the fragment amplified with PoABI5-GFP-F/R primers lacked a stop codon. Subsequently, genomic DNA was extracted from *P. ostii* using the CTAB method and used as a template to amplify the target fragment with ProPoFAD3-GUS-F/R primers. The resulting PCR product was then cloned into the pBI121 vector. All PCR primers used in this study are listed in Table 1.

### 2.4. Agrobacterium Transformation, Cultivation, and Preparation of Infiltration Suspension

Given the versatility of GV3101 and its demonstrated efficacy in the transient transformation of *P. ostii* tissues in prior studies, this work employed the GV3101 strain for Agrobacterium-mediated transformation [10,22,23]. The Agrobacterium strain GV3101 competent cells (Weidi Biotech, Shanghai, China), stored at −80 °C, were thawed on ice. Subsequently, 1 μL of target plasmids was added, and the mixture was incubated on ice for 5 min, quick-frozen in liquid nitrogen for 5 min, and then incubated at 37 °C for 5 min. After a further 5 min incubation on ice, 700 μL of fresh Yeast Extract Peptone (YEP) liquid medium was added. The culture was incubated with shaking at 28 °C and 200 rpm for 3 h. Subsequently, 200 μL of the bacterial culture was plated on a YEP agar plate containing rifampicin (25 mg/L), gentamicin (50 mg/L), and kanamycin (50 mg/L). The plate was then incubated at 28 °C in the dark for 48 h.

A single colony was picked and inoculated into 200 µL YEP liquid medium with 50 µg/mL kanamycin, 50 µg/mL gentamicin, and 25 µg/mL rifampicin. It was incubated at 28 °C with shaking for 5 h. Then, the bacterial culture was diluted and regrown at 28 °C, 200 rpm for 10–12 h until the target OD was reached. Cells were collected by centrifugation at 6000 rpm for 10 min at 4 °C, then resuspended in AS solutions (10 mM MgCl_2_ + 10 mM MES + AS) at varying concentrations (Table 2).

### 2.5. Infiltration of the P. ostii Seedlings

The 50 mL sterile syringe was taken, and the injection port was closed, after which 20 mL of Agrobacterium suspension and an appropriate number of embryo seedlings were added in turn. The syringe was then extracted for negative pressure treatment. The duration of each treatment was set at five seconds, and negative pressure treatment was administered in accordance with the target number (Table 2). Subsequently, the seedlings and Agrobacterium suspension were transferred into a tissue culture bottle and shaken at 100 rpm at 28 °C for the target duration. Following infection, the seedlings were excised, air-dried on sterile filter paper, and inoculated onto WPM medium that had been supplemented with 30 g/L sucrose, 0.5 mg/L 6-BA, 1.0 mg/L GA_3_, and 200 μmol/L AS (pH = 5.6). The seedlings were cultivated in the dark at 24 ± 2 °C for a 24 h period, then co-cultured under a 16 h light (2000 Lx)/8 h dark photoperiod for 2 days. Detailed information is provided in Table 2.

### 2.6. Orthogonal Experimental Design for Agrobacterium-Mediated Transient Genetic Transformation

In Agrobacterium-mediated transient genetic transformation, multiple factors can impact transformation efficiency. To identify the optimal infection conditions, this study conducted orthogonal experiments. Four factors were selected, each having three levels. Using the L9(3^4^) orthogonal array as a template, the fifth factor was sequentially assigned to the last column at levels 1, 2, and 3, generating nine experimental treatments. Each treatment consisted of 30 embryogenic seedlings with three biological replicates (Table 2).

### 2.7. GUS Histochemical Analysis and Enzyme Activity Assay

The embryogenic seedlings that had undergone co-cultivation were rinsed three times with sterile water and then placed in GUS staining solution for staining at 37 °C in the dark for 18 h. After staining, the seedlings were transferred to 75% ethanol for decolorization for 5 h, followed by a further decolorization in 95% ethanol for 10 h. The ethanol could be changed 2–3 times until all pigments were removed. The seedlings were photographed, and the transient expression rate of GUS was recorded and calculated according to the following criteria:

The GUS transient expression was categorized into three levels (GUS-1, GUS-2, and GUS-3) based on the staining intensity and area in the embryogenic seedlings, to compare and evaluate the effectiveness of different infection condition combinations. The ratio of stained area to total seedling area was quantified using ImageJ 1.53a. GUS-1: Light staining or obvious GUS spots, but with an area less than 50% of the total seedling area. GUS-2: Distinct staining with an area ≥ 50% of the total seedling area. GUS-3: Very distinct deep blue staining with an area > 50% of the total seedling area.

The transient expression rates of GUS were calculated as follows:

GUS transient expression rate (%) = Number of seedlings showing GUS staining/Total number of stained seedlings × 100; GUS-1/2/3 grade rate (%) = Number of seedlings showing GUS-1/2/3 level staining/Total number of stained seedlings × 100

GUS activity was quantified fluorometrically. Tissue samples were flash-frozen in liquid nitrogen, homogenized in extraction buffer, and centrifuged at 12,000× *g* for 15 min to collect the supernatant. These were utilized for GUS assays, with MUG serving as the substrate. Following the incubation period, the reactions were halted, and the GUS activity was measured at 365/455 nm using a fluorometer. The specific activity was expressed as nmol of 4 MU/mg protein·min^−1^ [39].

### 2.8. GFP Fluorescence Observation

After co-cultivation, embryogenic seedlings were examined for GFP fluorescence using a portable UV analyzer under total darkness, and images were captured. Fluorescent tissues were embedded in agarose gel and sectioned with a vibratome (VT1000S, Leica, Berlin, Germany) to generate temporary slides. Subsequently, GFP fluorescence was visualized under a confocal laser scanning microscope (CLSM, TCS SP8, Leica, Berlin, Germany) at an excitation wavelength of 488 nm and an emission wavelength range of 500–560 nm.

### 2.9. Virus-Induced Gene Silencing of PoABI5

The pTRV2:GFP and pTRV2:PoABI5 constructs were transformed into Agrobacterium tumefaciens strain GV3101. Single colonies were cultured overnight at 28 °C in YEP medium supplemented with rifampicin (25 mg/L), gentamicin (50 mg/L), and kanamycin (50 mg/L) until reaching an OD_600_ of 1.0. Bacterial cells were harvested by centrifugation at 6000 r/min for 10 min and resuspended in an equal volume of infiltration buffer (10 mM MgCl_2_, 10 mM MES, 200 μM AS, pH = 5.6). Two separate mixtures were prepared: Equal volumes of pTRV1 and pTRV2:GFP suspensions; Equal volumes of pTRV1 and pTRV2:PoABI5 suspensions. Each mixture was incubated at 28 °C in the dark for 3–4 h. Embryogenic seedlings were subjected to vacuum infiltration using a 50 mL syringe with the needle removed. A volume of 20 mL of the Agrobacterium mixture and seedlings were placed in the syringe, and a vacuum of −0.8 MPa was applied six times for 5 s each. The seedlings and suspension were then transferred to a tissue culture flask and co-cultivated at 28 °C with gentle shaking (100 rpm) for 2 h. Following incubation, seedlings were blotted dry on sterile filter paper and transferred to WPM basal medium supplemented with 30 g/L sucrose, 0.5 mg/L 6-BA, 1.0 mg/L GA_3_, and 200 μM AS (pH 5.6). Samples were first incubated in the dark at 24 ± 2 °C for 1 day, followed by a 16 h light (2 × 10^3^ lx)/8 h dark photoperiod for 2 days before analysis.

### 2.10. FA Extraction and Analysis

The present study methodically extracts the fatty acids from the seedlings of *P. ostii*, according to the method described by Ma et al. [40]. In summary, 100 mg of seedlings were placed into 4 mL of a methanol solution containing 2.5% (*v*/*v*) sulfuric acid, and the mixture was heated at 80 °C for 2 h. Following the transmethylation reaction, 2 mL of 0.9% NaCl and 1 mL of hexane were added for gas chromatography (GC) analysis. The analysis of FAs by GC was conducted in accordance with the established protocol [25].

## 3. Results

### 3.1. Optimal Germination Medium Screening for P. ostii Embryos

**Figure 2:**
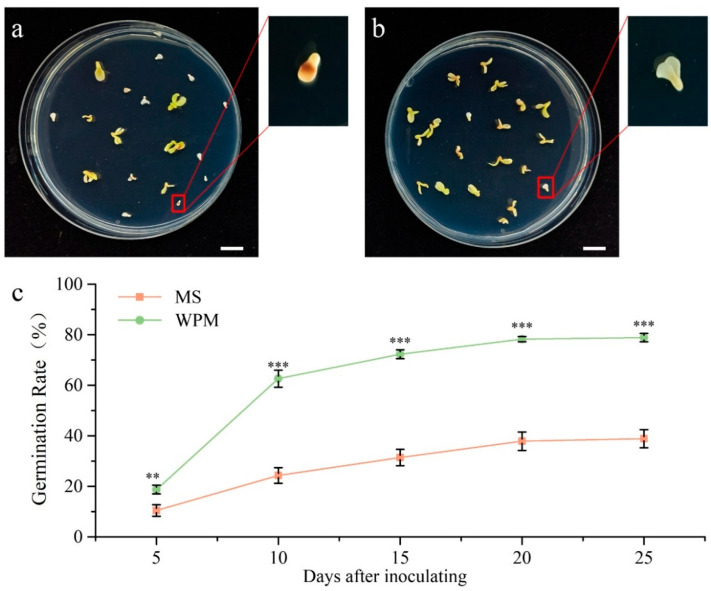
**Screening of optimal germination medium for *P. ostii* embryos.** (**a**). Germination status of *P. ostii* embryos on MS 15 days after inoculation, Bars = 1 cm; (**b**). Germination status of *P. ostii* embryos on WPM 15 days post-inoculation, Bars = 1 cm; (**c**). Time-course of embryo germination rates on MS and WPM media. A *t-test* was performed between all biological replicates and the control sample. Samples with significant differences are marked with asterisks (** *p* < 0.01, *** *p* < 0.001).

Mature seeds were harvested, air-dried, and used as experimental materials in this study. A refined embryo germination protocol for *P. ostii* seeds was employed to obtain in vitro embryo-derived seedlings for use in the transient transformation system (Figure 1). To further optimize the system, it is necessary to determine the optimal medium for initiating embryo germination in *P. ostii*. Based on previous studies, the germination rates in MS and WPM were evaluated [17,30]. Non-germinated embryos exhibited brownish-yellow spotting and lacked significant cotyledon expansion (Figure 2a), whereas successfully germinated ones appeared creamy white with expanded cotyledons (Figure 2b). Germination rates were regularly recorded (Figure 2c), showing a substantial difference as early as 5 days after inoculation (10.44% in MS vs. 18.7% in WPM), with the most conducive germination period occurring at 5–10 days, when 24.33% (MS) and 62.59% (WPM) of seeds germinated. Thereafter, germination levels stabilized at 38.88% (MS) and 78.88% (WPM, 2.03-fold higher) by day 20, with no significant differences observed between 20 and 25 days. Consequently, WPM was selected as the optimal medium for initiating embryo germination in *P. ostii* for subsequent experiments.

### 3.2. Orthogonal Optimization of Agrobacterium-Mediated Transient Transformation

**Figure 3:**
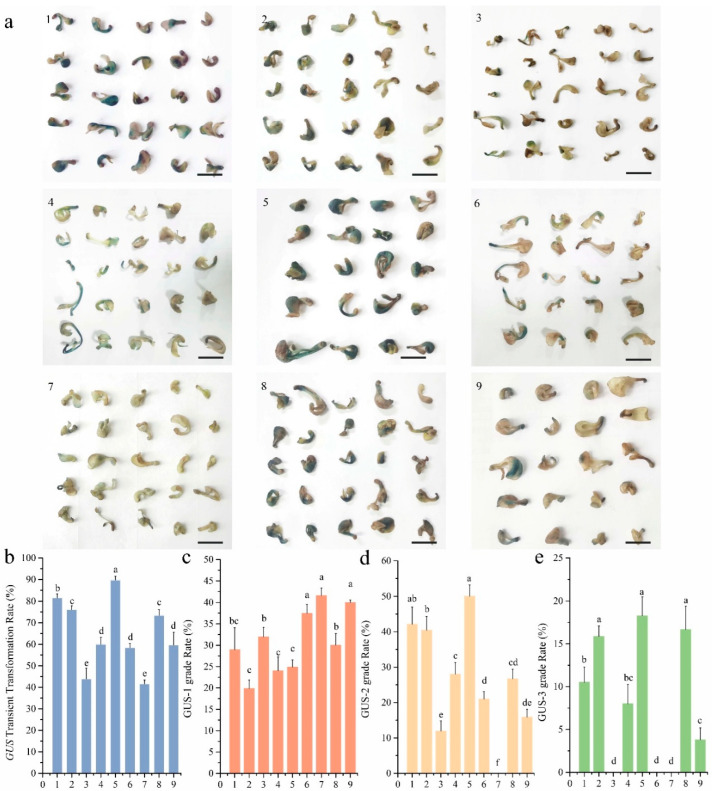
**Orthogonal optimization of Agrobacterium-mediated transient transformation.** (**a**). GUS staining phenotypes of seedlings, 1–9 represent nine experimental treatment groups designed based on the orthogonal experiment, Bars = 1 cm; (**b**). Transient transformation rate of GUS; (**c**). GUS-1 transient expression rate of seedlings; (**d**). GUS-2 transient expression rate of seedlings; (**e**). GUS-3 transient expression rate of seedlings. Error bar = standard deviation. Different letters above each bar indicate significant differences according to one-way ANOVA with Duncan’s multiple comparisons test (*p* < 0.05).

In this study, a homologous protocol has been developed for the in vitro embryo-derived seedlings of *P. ostii*, drawing on the established transient transformation system of *P. lactiflora* (Figure 3a). To optimize Agrobacterium-mediated transient transformation efficiency, a L9(3^4^) orthogonal array was used to investigate four three-level factors—Agrobacterium suspension OD_600_, AS concentration, vacuum infiltration cycles, and infection duration, yielding nine treatment combinations (Table 2). The transformation efficiency of each group was assessed using GUS staining, with the results of the partial staining for the nine experimental groups presented in Figure 2b. The findings of the analysis demonstrated that Treatment 5 exhibited the highest GUS transient expression rate at 89.74%, with Treatment 1 following closely behind at 81.59% (Figure 3b). A further hierarchical comparison of GUS staining intensity demonstrated that for the strongest staining category (GUS-3), Treatments 2, 5, and 8 exhibited optimal performance, with GUS-3 proportions of 15.85%, 18.26%, and 16.67%, respectively (Figure 3e). Within the GUS-2 category, Treatments 1 and 5 exhibited the highest proportions, at 42.1% and 50%, respectively (Figure 3d). In the category of weakest staining (GUS-1), treatments 6, 7, and 9 exhibited higher proportions of 37.43%, 41.58%, and 39.96%, respectively (Figure 3c). Collectively, Treatment 5 displayed the highest GUS transient expression rate and the highest proportions in both GUS-2 and GUS-3 categories. The following protocol has been determined to be the most effective Agrobacterium-mediated transient transformation: the OD_600_ of the culture was measured to be 1.0, AS concentration in the resuspension buffer was 200 μM, the negative pressure treatment was performed six times, and the infection duration was 2 h.

### 3.3. Impact of Growth Status of In Vitro Embryo-Derived Seedlings in P. ostii on Agrobacterium Infection Efficiency

**Figure 4:**
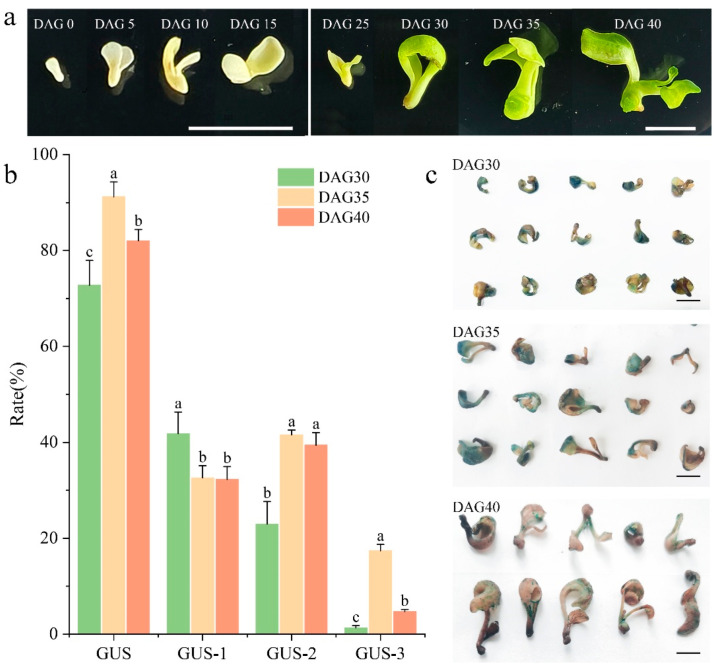
**Effects of in vitro embryo-derived seedling Growth Status on Agrobacterium Infection Efficiency.** (**a**). Morphological stages of in vitro embryo-derived seedling, Bars = 1 cm; (**b**). GUS transient transformation rate efficiency across seedling growth states, Error bar = standard deviation. Different letters above each bar indicate significant differences according to one-way ANOVA with Duncan’s multiple comparisons test (*p* < 0.05); (**c**). GUS Staining phenotypes of in vitro embryo-derived seedlings, Bars = 1 cm.

Given the close correlation between infection efficiency and plant growth status, the effect of in vitro embryo-derived seedling growth status on Agrobacterium infection efficiency was further investigated. Germinated seedlings were monitored from the inoculation day, designated as day after germination (DAG), with growth phenotypes documented via periodic observations and photography at 5-day intervals (Figure 4a). Before DAG25, seedlings showed a creamy white hue and sluggish vegetative growth, while chlorophyll accumulation initiated greening with accelerated growth at DAG25-30; callus formation first appeared at the base by DAG35, followed by true leaf emergence at DAG40, after which seedlings entered a senescence phase (growth arrest and yellowing) post-DAG40. Based on these observations, DAG30-40 seedlings were identified as optimal for Agrobacterium-mediated transient transformation.

Previously, DAG30 seedlings were selected as experimental material. To assess stage-specific infection efficiency variations, DAG30, 35, and 40 seedlings were infected using pre-identified optimal Agrobacterium parameters. GUS staining phenotypes were analyzed (Figure 4c), revealing that DAG35 seedlings exhibited the highest transient expression rate (91.23%), with 41.57% showing moderate (GUS-2) and 17.39% strong (GUS-3) staining, significantly higher than DAG30 and DAG40 counterparts (Figure 4b). Comparative analysis further showed DAG40 outperformed DAG30 in GUS staining efficiency. Collectively, these results identified DAG35 as the optimal developmental stage for *P. ostii* in vitro embryo-derived seedlings, and TTAES was established following the optimization of Agrobacterium infection parameters and determination of the optimal stage.

### 3.4. Elucidation of TTAES for Assessing Transcription Factor-Mediated Target Gene Promoter Activation

**Figure 5:**
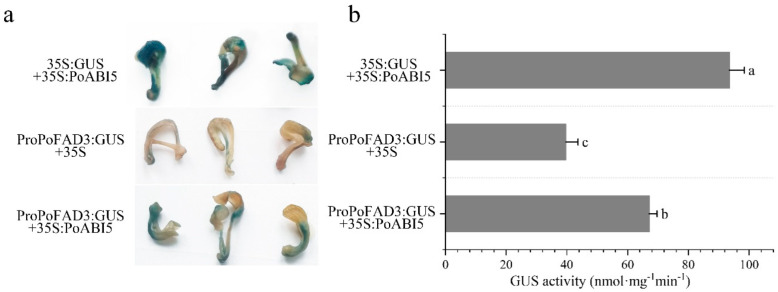
**Transcriptional activation of the PoFAD3 promoter by PoABI5 in *P. ostii.*** (**a**): GUS staining elucidation of *PoFAD3* promoter activation by PoABI5; (**b**): GUS enzyme activity assay for *PoFAD3* promoter transcription induced by PoABI5, Error bar = standard deviation. Different letters above each bar indicate significant differences according to one-way ANOVA with Duncan’s multiple comparisons test (*p* < 0.05).

To validate these results, TTAES was employed to assess the activation of target gene promoters mediated by transcription factors. The extant evidence demonstrates that PoABI5 has the capacity to upregulate *PoFAD3*, thereby contributing to the regulation of ALA synthesis in *P. ostii* seeds [22]. In this study, *PoABI5* and the *PoFAD3* promoter (ProPoFAD3) were successfully cloned into the pCAMBIA1300 and pBI121 vectors, respectively. TTAES was performed to assess GUS reporter expression and enzymatic activity across a range of experimental conditions. It has been demonstrated that the positive control group (35S:GUS + 35S:PoABI5) exhibits optimal GUS staining outcomes (Figure 5a) and the most elevated levels of GUS enzyme activity (Figure 5b), with a value of 93.55 nmol·mg^−1^·min^−1^. In comparison with the negative control group (ProPoFAD3:GUS + 35S), the experimental group (ProPoFAD3:GUS + 35S:PoABI5) demonstrated a more pronounced level of GUS staining (Figure 5a), with GUS enzyme activity reaching 67.13 nmol·mg^−1^·min^−1^, which was 1.69 times higher than that of the negative control group. This finding suggested that PoABI5 significantly activates the transcriptional activity of *PoFAD3* (Figure 5b). This finding was consistent with previous results [22], indicating that TTAES can be applied to verify the activation of target gene promoters by transcription factors.

### 3.5. Elucidation of TTAES as a GFP-Marked System for Gene Function Analysis

**Figure 6:**
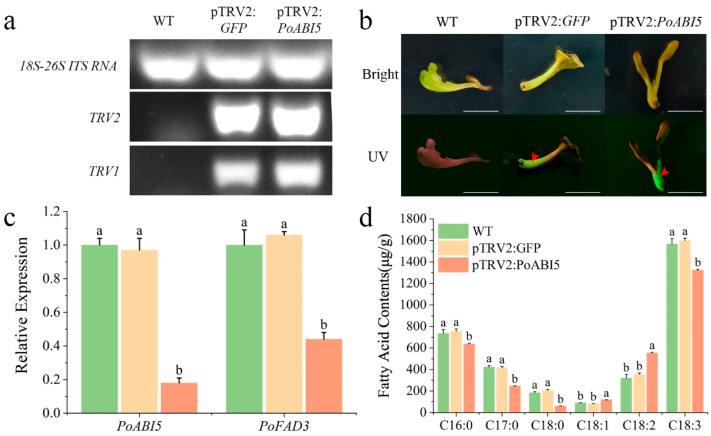
**Elucidation of *PoABI5* silencing via VIGS in vitro seedlings of *P. ostii*.** (**a**). Semi-quantitative RT-PCR analysis of *PoABI5* VIGS silencing; (**b**). UV imaging of seedlings after *PoABI5* VIGS silencing, Bars = 1 cm; (**c**). qRT-PCR quantification of *PoABI5* silencing efficiency; (**d**). Fatty acid composition changes in seedlings. Error bar = standard deviation. Different letters above each bar indicate significant differences according to one-way ANOVA with Duncan’s multiple comparisons test (*p* < 0.05).

Considering the irreversible damage of GUS staining to experimental materials, GFP tagging is commonly used for transformation detection in gene function elucidation [31,41]. TTAES of *P. ostii* was evaluated through two experiments using GFP as a tag and employing TRV-mediated VIGS and transient overexpression methods. The VIGS technique was employed to knock down *PoABI5* expression. Following the conclusion of the treatment, a period of one week was allowed to elapse before the plants were subjected to visualization under a portable UV lamp. The untreated wild-type (WT) sample displayed no fluorescence, whereas both pTRV2:GFP and pTRV2:PoABI5 tissues exhibited green fluorescence, indicating successful GFP protein expression and confirming effective agroinfiltration (Figure 6b). The efficiency of the VIGS system was validated by conducting semi-quantitative analysis of the fluorescent tissues. The presence of both *TRV1* and *TRV2* transcripts in the pTRV2:GFP and pTRV2:PoABI5 samples confirmed successful viral vector delivery (Figure 6a). qRT-PCR analysis revealed that *PoABI5* expression in the pTRV2: PoABI5 sample was reduced to 0.19-fold relative to the pTRV2: GFP sample, thereby demonstrating significant gene silencing (Figure 6c). Concomitantly, *PoFAD3* expression in pTRV2:PoABI5 was downregulated to 0.42-fold compared to the control (Figure 6c). Fatty acid content of WT, pTRV2:GFP, and pTRV2:PoABI5 tissues demonstrated a significant decrease in C18:3 (α-linolenic acid) and an increase in C18:2 (linoleic acid) in the silenced line (Figure 6d). These findings demonstrated the efficacy of TTAES in facilitating gene function elucidation of *P. ostii* through the implementation of GFP-tagged VIGS assays. This system provided a robust platform for gene function elucidation in *P. ostii*.

**Figure 7:**
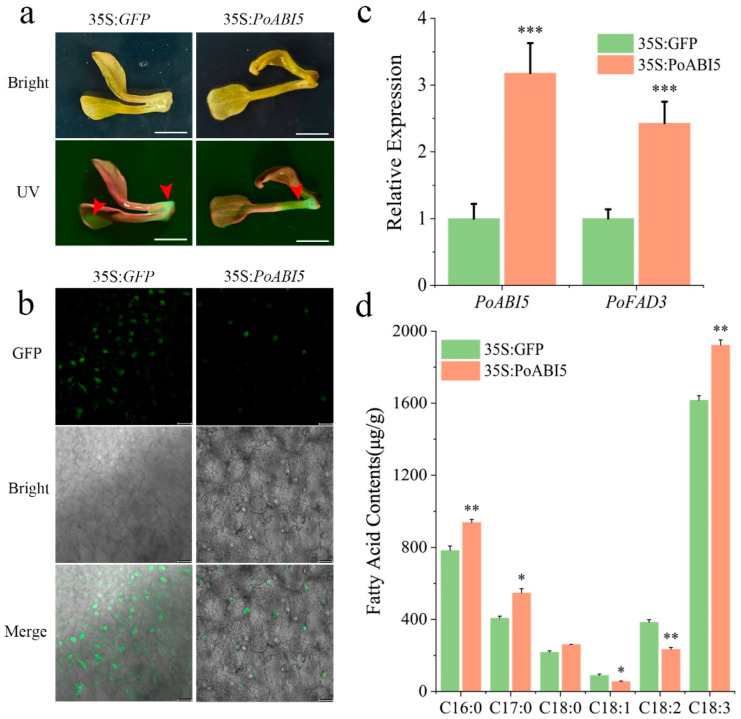
**Elucidation of PoABI5 overexpression in seedlings of *P. ostii.*** (**a**). UV fluorescence imaging of *PoABI5* overexpressing seedlings, Bars = 1 cm; (**b**). Confocal microscopy analysis of *PoABI5* overexpressing seedlings; (**c**). qRT-PCR quantification of *PoABI5* overexpression efficiency; (**d**). Fatty acid composition changes in *PoABI5* overexpressing seedlings. Samples with significant differences are marked with asterisks (* *p* < 0.05, ** *p* < 0.01, *** *p* < 0.001).

*PoABI5* was cloned into the pCAMBIA2300-GFP vector for TTAES. Within one week after transformation, the plants were visualized under a portable UV lamp, and the resulting green fluorescence confirmed the successful transient expression of both 35S:GFP and 35S:PoABI5 (Figure 7a). Subsequent analysis using laser confocal microscopy further validated that GFP signals were detected exclusively in the 35S:GFP and 35S:PoABI5 samples, a finding that supported the efficacy of the transient expression process (Figure 7b). qRT-PCR analysis revealed that PoABI5 expression in the 35S:PoABI5 group was significantly upregulated compared to the 35S:GFP control, with *PoFAD3* expression in the overexpressing line reaching 2.43-fold the level of the control group, as presented (Figure 7c). The study revealed that the expression of *PoABI5*, which was observed as being significantly increased, was accompanied by a transient increase in the concentration of C18:3, whilst concurrently there was a decrease in the level of C18:2 (Figure 7d). Altogether, these results demonstrated that TTAES of *P. ostii* is effective for elucidating GFP-tagged gene function, thereby offering a robust platform for molecular research in this species.

## 4. Discussion

Seed germination in *P. ostii*, strictly regulated by morphophysiological dormancy, requires harvesting and sowing at dormancy-specific optimal timing to avoid a significant reduction in germination rates [42]. Previous studies investigating germination requirements using mature embryos as explants employed modified MS medium to examine the effects of different hormone concentrations on embryo germination and identified 0.5 mg/L BA + 1.0 mg/L GA_3_ as the optimal treatment [43]. Consequently, this hormone combination was adopted in our study. However, previous research utilized exclusively MS as the basal medium for embryonic germination. To evaluate the impact of different basal media, a comparison was made between MS and WPM. Studies on embryo maturation have shown that WPM promotes higher adventitious bud induction than MS during subsequent shoot development [16,37,44]. Our results demonstrate that, under identical hormone conditions, WPM resulted in a significantly higher embryo germination rate (Figure 2c). This phenomenon may be attributed to the composition of WPM, which is characterized by lower overall nutrient levels, specifically reduced nitrogen and potassium concentrations. These lower nutrient levels may better suit the growth requirements of woody plants. In addition, the relatively higher calcium content of WPM may enhance cell wall stability and stress tolerance [38]. In contrast, MS medium, distinguished by its high nitrate concentration and extensive use in plant culture, exhibits elevated salt ion levels. This inherent property has been demonstrated to exacerbate explant browning [13,16], a phenomenon that is consistent with the observed browning and yellowing of non-germinated embryos on MS medium in the present study (Figure 2a).

Agrobacterium-mediated transformation efficiency is influenced by a number of factors, including bacterial density (OD_600_) [17,45], AS concentration [18,46], the number of vacuum infiltration cycles [45,47], infection duration [17,48], and the co-culture period [49]. In the present study, a combination of parameters that have been demonstrated to be optimal was established. The experimental conditions comprised an Agrobacterium density at an OD_600_ of 1.0, AS supplementation at a rate of 200 µmol/L in the bacterial suspension, six negative pressure treatments, and an infection time of 2 h (Figure 3). It is evident that there is variability in the optimal Agrobacterium density (OD_600_) across peony species and explant types. For instance, studies report OD_600_ = 1.0 for cut flowers of Itoh peony ‘Bartzella’ [4], OD_600_ = 0.6 for flower buds of tree peony (*P. suffruticosa*) ‘Yulouchun’ [24], and OD_600_ = 1.5 for tuberous roots of *P. lactiflora* ‘Da Fugui’ [50]. The findings of the present study identified OD_600_ = 1.0 as optimal. Suboptimal bacterial density may compromise transformation efficiency due to insufficient bacterial-explant contact, while excessive concentrations can induce explant browning or necrosis, thereby reducing transformation frequency [20]. In a similar manner, the duration of infection and co-culture has been demonstrated to exert an influence on efficiency by modulating the period of interaction between Agrobacterium and the explant. While the duration of contact is known to have a positive effect on transformation rates, prolonged exposure has been shown to cause explant damage, resulting in diminished transient expression efficiency [20,30]. As a phenolic virulence inducer, AS activates the vir gene region of Agrobacterium, thereby improving T-DNA transfer efficiency. However, explants exhibit substantial variation in their sensitivity to AS [51]. Our optimal AS concentration (200 µmol/L) aligns with numerous peony Agrobacterium-mediated transient transformation protocols [10,22,24,50,52].

Previous studies have shown that the developmental stage of plant tissues significantly influences the outcomes of transformation processes. Transient leaf transformation studies demonstrate that there is a decline in transformation efficiency that is correlated with an increase in leaf age [53,54]. The maturation process is characterized by an increase in vein density, resulting in the partitioning of the mesophyll apoplast. Consequently, upon traversing the epidermal barrier, the bacterial suspension encounters restricted diffusion within the leaf, thereby diminishing its efficiency. In accordance with this phenomenon, the present study observed a decline in efficiency in later developmental stages of sterile tissue-cultured embryogenic seedlings, characterized by growth retardation, cotyledon senescence, and emergence of true leaves, mirroring the pattern seen in mature leaves. In the study of the instantaneous transformation of the strawberry, it was ascertained that infection at the fruit receptacle was difficult in the early stage of fruit firmness. The optimum infection period was identified as occurring before the rapid decrease in fruit hardness and the onset of red coloration [55]. This limitation can be attributed to reduced intercellular space and denser cell packing during the initial developmental stages, which physically impedes suspension diffusion and compromises transformation [53]. The lower transformation efficiency observed at DAG30 compared to DAG35 in the present system may be attributable to this developmental constraint.

Compared to existing Agrobacterium-mediated transient transformation systems for *P. ostii*, the TTAES approach offers distinct advantages. First, as a high-yielding woody oil crop, *P. ostii* produces substantially more seeds than other species and varieties in the same genus—mature 7-year-old plants yield up to 10 fruits and 200–500 seeds per plant, enabling the procurement of abundant material of the same genotype for functional validation [1,26,56]. Second, unlike experiments requiring buds, leaves, or floral tissues with strict seasonal/temperature constraints, TTAES utilizes storable seeds that permit year-round seedling cultivation within 12 months post-harvest [10,22,25,57]. Finally, TTAES employs GFP as a reporter for non-destructive transgenic identification while laying a foundation for further exploring the application of this system in subcellular localization, BiFC, or dual-label imaging.

To further validate TTAES, we applied it to analyze the regulatory relationship between a target transcription factor and its cognate gene. ALA, an essential polyunsaturated fatty acid for humans, is a focus of ongoing research on its biosynthetic pathway. It has been demonstrated that ABA regulates lipid synthesis-related gene transcription by activating ABI5 [5,22,58]. Specifically, PoABI5 is responsible for activating the transcription of *PoFAD3*, thus promoting ALA synthesis [22]. While the transcriptional activation of the *PoFAD3* promoter by PoABI5 was initially validated in a tobacco heterologous system, we further confirmed this interaction in *P. ostii* using TTAES. It was determined that PoABI5 activated the *GUS* driven by the *PoFAD3* promoter, resulting in a 1.69-fold increase in GUS enzyme activity relative to the negative control (Figure 5b). These findings are consistent with the conclusions drawn from the tobacco system [22]. This finding enables the verification of the relationship between the transcription factors of *P. ostii* and the target gene promoters in the native system. Consequently, this ensures the accuracy and reliability of the experimental results.

In gene function studies, phenotypic changes induced by gene silencing or overexpression are often assessed using reporter tags. Although GUS tags offer high sensitivity and stability, GUS staining or activity assays inevitably damage experimental materials, compromising subsequent phenotypic analysis [41]. In contrast, GFP enables non-destructive in vivo observation, making it preferable for elucidating transient silencing and overexpression [31,41]. In this study, we integrated GFP into TTAES and performed VIGS-mediated silencing (pTRV2 vector) and transient overexpression of *PoABI5.* Expression levels of its target gene (*PoFAD3*) and ALA content were quantified by qRT-PCR and GC-MS, respectively. This approach provides a more rapid and non-destructive alternative to traditional GUS-based methods for gene function elucidation in *P. ostii*.

## 5. Conclusions

This study established an efficient transient transformation system, TTAES in *P. ostii*, which overcomes seasonal constraints in gene function elucidation by optimizing embryo germination protocols to enable year-round in vitro seedling propagation. Orthogonal experimental design identified optimal parameters: OD_600_ = 1.0, 200 μM AS, six negative pressure treatments, and 2 h infection duration, which achieved peak transformation efficiency in vitro embryo-derived seedlings of *P. ostii* 35 days after germination. Using GUS and GFP reporter systems, the platform confirmed transcription factor–promoter interactions and enabled VIGS and overexpression analyses, demonstrating its utility for functional genomics. This system eliminates seasonal restrictions, accelerates molecular breeding workflows, and provides a robust framework for elucidating gene functions in *P. ostii*, advancing its development as a dual-purpose medicinal and oilseed crop. However, as a transient transformation system, TTAES exhibits phenotypic variability due to transformation efficiency fluctuations. Moreover, because phenotypic assessment commences as early as 3 days post-transformation, it is unsuitable for functional validation requiring prolonged transgene expression to manifest phenotypes. For CRISPR-mediated knockout or stable transgenic line generation, optimization of seedling rooting protocols and post-transformation screening regimes remains necessary.

## Figures and Tables

**Table 1:** Primers used in this study.

Primer Name	Primer Sequence (5′-3′)	Annotation
ProPoFAD3-GUS-F	ACCATGATTACGCCAAGCTTTGTTCGTCTCGTCGTCCTCTTC	Vector Construction
ProPoFAD3-GUS-R	GACTGACCACCCGGGGATCCTTGGCCTTCGGTTCACAGAT	
35S:PoABI5-F	ACGGGGGACGAGCTCGGTACCATGGTTGTTCCTGAGTCCGAAA	
35S:PoABI5-R	GCTCTGCAGGTCGACTCTAGATCAAAAAGGGGAACTCAAAGTCC	
PoABI5-pTRV2-F	GAAGGCCTCCATGGGGATCCTAAACCAGCGCGGTTATCCA	
PoABI5-pTRV2-R	GGACATGCCCGGGCCTCGAGGGATTGTCTTCCCAATGATGCA	
PoABI5-GFP-F	ACGGGGGACGAGCTCGGTACCATGGTTGTTCCTGAGTCCGAAA	
PoABI5-GFP-R	CTTGCTCACCATGGTGTCGACAAAAGGGGAACTCAAAGTCCTCC	
PoABI5-RT-F	TGGAAGCAGAGCTGAACCAG	RT-PCR /qRT-PCR
PoABI5-RT-R	CGAACACCTCAAAAAGGGGA	
PoFAD3-RT-F	GGCAGCCACATTTCCGTCTT	
PoFAD3-RT-R	AGTAGAGTGGCCAAACAGCC	
TRV1-RT-F	CAGTCTATACACAGAAACAGA	
TRV1-RT-R	GACGTGTGTACTCAAGGGTT	
TRV2-RT-F	GGCTAACAGTGCTCTTGGTG	
TRV2-RT-R	GTATCGGACCTCCACTCGC	
18S-26S ITS RNA-F	ACCGTTGATTCGCACAATTGGTCATCG	
18S-26S ITS RNA-R	TACTGCGGGTCGGCAATCGGACG	

**Table 2:** Orthogonal experimental design for Agrobacterium-mediated transient transformation.

Test Number	OD_600_	AS Concentration (μmol·L^−1^)	Number of Negative Pressure	Infection Time (h)
1	0.8	150	2	2
2	0.8	200	4	4
3	0.8	250	6	8
4	1.0	150	4	8
5	1.0	200	6	2
6	1.0	250	2	4
7	1.2	150	6	4
8	1.2	200	2	8
9	1.2	250	4	2

## Data Availability

All data generated or analyzed during this study are included in this published article.
